# UAVs-FFDB: A high-resolution dataset for advancing forest fire detection and monitoring using unmanned aerial vehicles (UAVs)

**DOI:** 10.1016/j.dib.2024.110706

**Published:** 2024-07-03

**Authors:** Md. Najmul Mowla, Davood Asadi, Kadriye Nur Tekeoglu, Shamsul Masum, Khaled Rabie

**Affiliations:** a Alparslan Türkeş Science and Technology University, Adana 1250, Turkey; b Hasan Kalyoncu University, Gaziantep 27100, Turkey; c University of Portsmouth, Portsmouth PO1 3DJ, United Kingdom; d Manchester Metropolitan University (MMU), Manchester M15GF, United Kingdom

**Keywords:** Forest fire, Unmanned aerial vehicles, Deep Learning, Machine learning, Convolutional neural networks, Detection and classification

## Abstract

Forest ecosystems face increasing wildfire threats, demanding prompt and precise detection methods to ensure efficient fire control. However, real-time forest fire data accessibility and timeliness require improvement. Our study addresses the challenge through the introduction of the Unmanned Aerial Vehicles (UAVs) based forest fire database (UAVs-FFDB), characterized by a dual composition. Firstly, it encompasses a collection of 1653 high-resolution RGB raw images meticulously captured utilizing a standard S500 quadcopter frame in conjunction with a RaspiCamV2 camera. Secondly, the database incorporates augmented data, culminating in a total of 15560 images, thereby enhancing the diversity and comprehensiveness of the dataset. These images were captured within a forested area adjacent to Adana Alparslan Türkeş Science and Technology University in Adana, Turkey. Each raw image in the dataset spans dimensions from 353 × 314 to 640 × 480, while augmented data ranges from 398 × 358 to 640 × 480, resulting in a total dataset size of 692 MB for the raw data subset. In contrast, the augmented data subset accounts for a considerably larger size, totaling 6.76 GB. The raw images are obtained during a UAV surveillance mission, with the camera precisely angled a -180-degree to be horizontal to the ground. The images are taken from altitudes alternating between 5 - 15 meters to diversify the field of vision and to build a more inclusive database. During the surveillance operation, the UAV speed is 2 *m/s* on average. Following this, the dataset underwent meticulous annotation using the advanced annotation platform, Makesense.ai, enabling accurate demarcation of fire boundaries. This resource equips researchers with the necessary data infrastructure to develop innovative methodologies for early fire detection and continuous monitoring, enhancing efforts to protect ecosystems and human lives while promoting sustainable forest management practices. Additionally, the UAVs-FFDB dataset serves as a foundational cornerstone for the advancement and refinement of state-of-the-art AI-based methodologies, aiming to automate fire classification, recognition, detection, and segmentation tasks with unparalleled precision and efficacy.

Specifications Table

**Table:** 

Subject	Unmanned Ariel Vehicles (UAVs), Computer Science, Aerospace, Environmental Engineering
Specific subject area	Artificial Intelligence, Computer Vision, Pattern Recognition, and Ecology
Type of data	RGB images
Data collection	The UAVs-FFDB datasets were acquired using a Raspberry Pi 4 camera integrated into the S500 multirotor UAVs platform. Raw images exhibit dimensions ranging from (353 × 314) to (640 × 480), while augmented images vary from (398 × 358) to (640 × 480). The dataset comprises four distinct classes: Pre-evening, forest condition, evening forest condition, pre-evening fire incident, and evening fire incident. Image acquisition occurred under diverse environmental conditions, encompassing variations in lighting such as daylight and evening settings.
Data source location	Adana Alperslan Turkes Science and Technology University, Adana, Turkey
Data accessibility	Repository name: Mendeley Data Data identification number: 10.17632/5m98kvdkyt.2 Direct URL to data: https://data.mendeley.com/datasets/5m98kvdkyt/2
Related research article	None

## 1 Value of the Data

• The UAVs-FFDB dataset significantly advances forest fire monitoring and management by offering high-quality, annotated imagery for training models capable of real-time data analysis. It addresses a crucial gap in existing datasets by providing comprehensive, high-resolution imagery for precise forest fire analysis, particularly in regions lacking adequate data. Unlike datasets especially sourced from internet images, this dataset includes data collected directly from real forest environments, enhancing its relevance and applicability.
• Researchers and practitioners in forestry, environmental science, and fire management can leverage this dataset to enhance early detection, monitoring, and response strategies for forest fires. It facilitates exploring solutions for improving fire detection and management, utilizing recent advancements in aerial monitoring systems to provide accurate insights into fire behavior for enhanced functional efficiency.
• The UAVs-FFDB dataset presents considerable possibilities for computer scientists and their domain specializing in UAV technology to design and validate state-of-the-art fire detection and classification architectures. The dataset exceeds the conventional binary classification of fire and no fire by presenting various classes, including pre-evening forest conditions, evening forest conditions, pre-evening fire incidents, and evening fire incidents. These precise classes provide crucial understandings of the effects of varying lighting and environmental conditions and early indicators preceding fire outbreaks on detection accuracy and predictive capabilities. Including such diverse training conditions enhances the robustness and reliability of UAV-based fire detection systems, thereby increasing their efficacy in real-world scenarios characterized by dynamic environmental conditions.
• The dataset also supports advancements in artificial intelligence (AI) based fire detection and monitoring processes, facilitating the advancement of image-based architecture approaches such as classification, real-time prediction, and image segmentation. Its broader applications extend to deep learning (DL) and computer vision (CV) tasks, benefiting fields outside fire detection by contributing to object segmentation, image analysis, and environmental monitoring. Overall, its availability fosters scientific progress and technological innovation, promoting more efficient and sustainable practices in forest management

## 2 Background

Applying machine learning (ML), DL, and CV models to improve fire detection in forest environments holds promise for prospective research endeavors [1]. Access to comprehensive datasets is crucial for developing efficient AI approaches in this domain [2]. However, obtaining real-time fire datasets presents a significant challenge due to the complexities of creating suitable experimental conditions [3]. The urgency of forest fires, with their potential for rapid spread, emphasizes the importance of timely detection mechanisms [4]. Existing literature indicates a scarcity of readily available real-time UAV datasets, primarily obtained from online repositories, still photos and processed using software tools such as Photoshop [[5], [6], [7]]. Early identification of forest fires is essential for pre-emptive measures and quick intervention [2]. Therefore, establishing a standardized real-time image dataset specifically designed for forest fire detection is imperative, enabling its integration into advanced AI architectures, including ML, DL, and CV methodologies. Our primary objective is to develop a standardized forest fire image dataset conducive to proficient classification, object detection, and recognition, aligning with the state-of-the-art frameworks in the AI domain, encompassing ML, DL, and CV.

## 3 Data Description

**Fig. 1:**
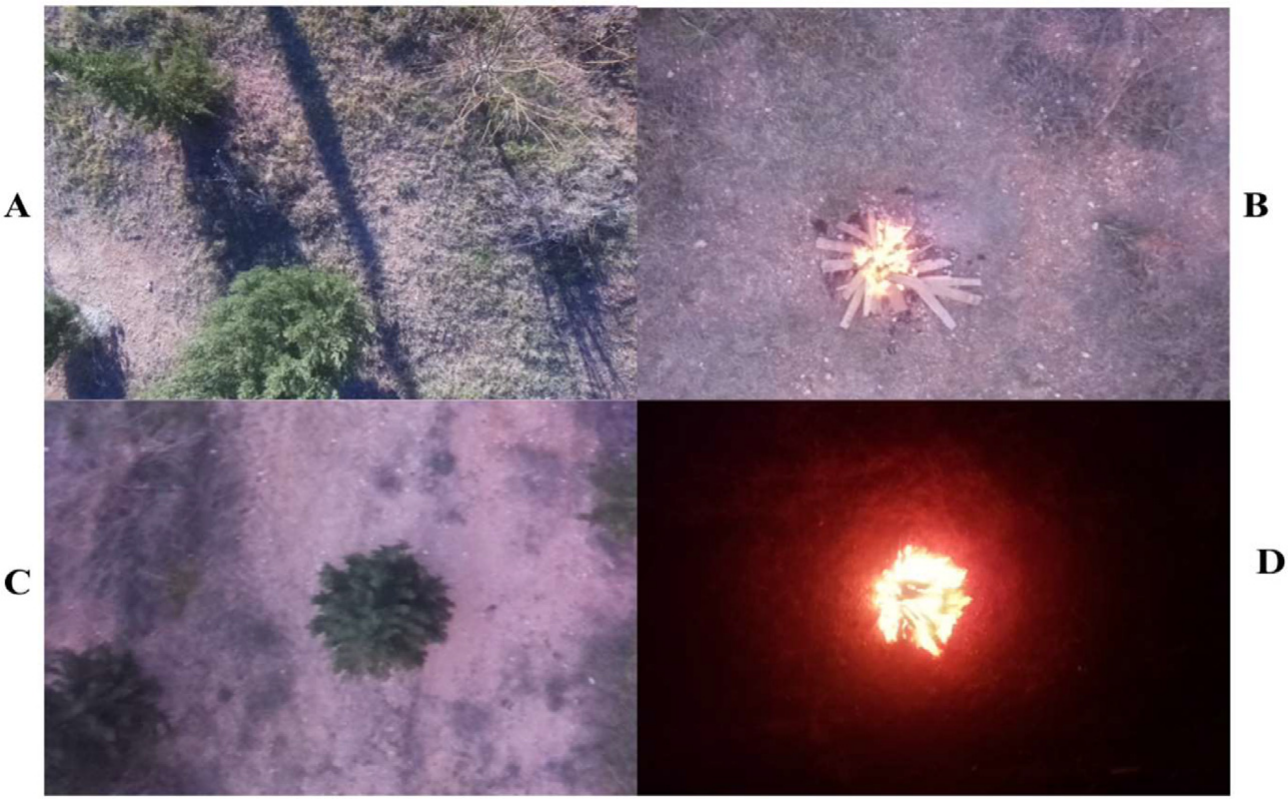
Sample of dataset (A) Pre-evening Forest condition (B) Pre-evening fire incident (C) Evening Forest condition (D) Evening fire incident.

The UAVs-FFDB dataset, comprising 1653 images collected by UAVs in the forest area of Adana Alperslan Turkes University, Saricam, Adana, Turkey, is a valuable resource for DL and advanced Computer Vision applications, particularly in forest fire detection and classification. It categorizes images into four classes: Pre-Evening Forest Condition, Evening Forest Condition, Pre-Evening Fire Incident, and Evening Fire Incident. Each of the 1653 RGB images within the dataset has dimensions of 640 × 480 and a 24-bit depth in PNG format. Fig. 1 illustrates sample images, while Table 1 provides additional details. Furthermore, Table 2 presents the distribution of classes along with their respective details. Besides, our dataset underwent rigorous annotation procedures to effectively target both object detection and recognition objectives, harnessing the sophisticated functionalities offered by the makesense.ai platform [8]. In this case we annotate in the classes where the fire is exists (Pre-evening fire incident and Evening fire incident classes). The resultant annotations were precisely archived in XML format, ensuring a balanced fusion and operational agility across successive phases of data processing and analytical endeavours.

**Table 1:** Details of UAVs-FFDB dataset.

Attributes	Parameter
Total number of data	1653
Number of classes	4
Dimension	353 × 314 to 640 × 480
Bit depth	24
Complete size	692 MB
Image format	PNG
Annotation format	XML

**Table 2:** UAVs-FFDB dataset: Class distribution and image quantification.

Classes	No of Images
Pre-evening Forest condition	222
Evening forest condition	286
Pre-evening fire incident	791
Evening fire incident	354

## 4 Experimental Design, Materials and Methods

### 4.1 Data Acquisition

**Fig. 2:**
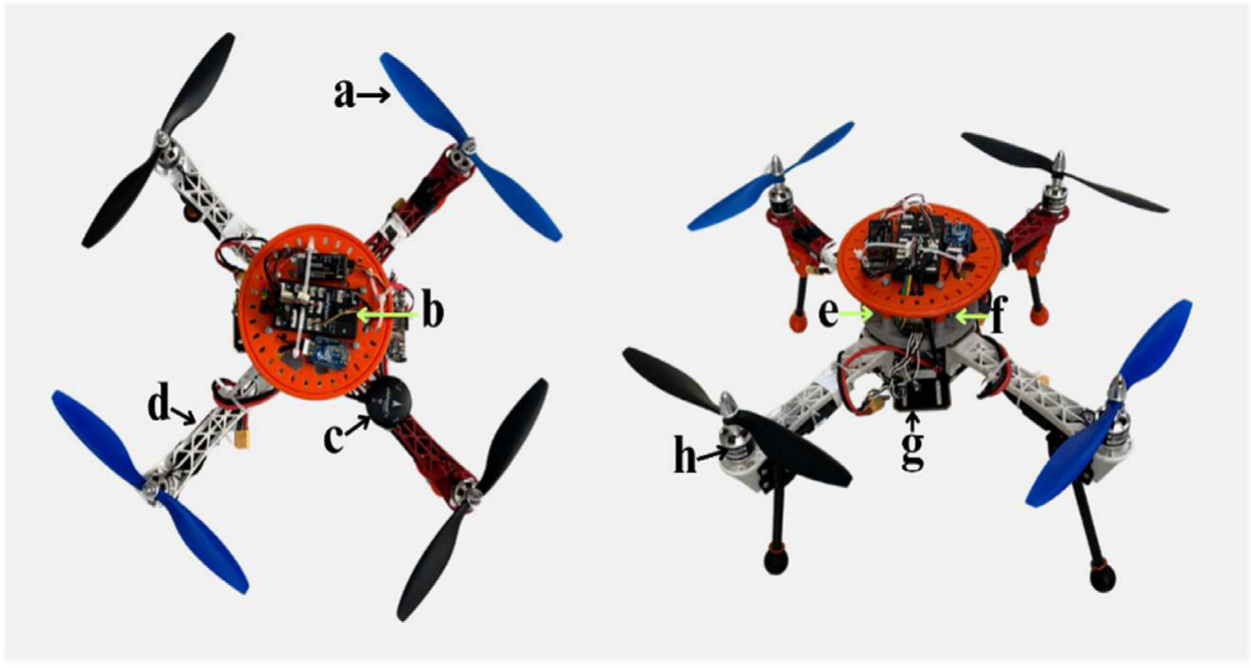
The hardware demonstration of the S500 quadrotor with its components (a) Propeller, (b) Flight Controller, (c) GPS Module (Pixhawk 4GPS), (d) Frame, (e) Onboard flight computer, (f) Battery, (g) Camera, (h) Motor.

The image acquisition system employed an S500 quadrotor UAV integrated with multiple components, comprising a RaspiCamV2, Xbee Telemetry board, Pixhawk4 flight controller, radio link module, Raspberry Pi 4, and supplementary tools such as a battery and voltage converter. These facilitated operations during the experimental phase by providing power to the Raspberry Pi and flight controller. Details regarding the use of UAVs for UAVs-FFDB data collection can be found in Table 3, while Fig. 2 illustrates the UAV configuration. The Raspberry Pi was the central storage unit for collected data, systematically accumulating images after each flight session. Following UAV activation and battery connection, a script executed on the Raspberry Pi initiated image capture at three frames per second. Subsequently, a separate script ensured the individual archiving of each image to prevent accidental overwriting. The imaging sensor was the Raspberry Pi Camera V2 (RaspiCamV2), mounted on the UAV frame with a downward inclination angle of -180°. Table 4 outlines the camera's characteristics, offering a detailed depiction of its features. This particular type of camera is capable of adjusting brightness and contrast, enabling it to provide satisfactory information about surface obstacles. Image capture operations were conducted at varying altitudes ranging from 5 to 15 meters to introduce diversity in perspective and enhance dataset inclusivity. The UAV maintained a consistent average operational velocity of 2 *m/s* throughout the image acquisition process.

**Table 3:** Multirotor UAV properties.

Components	Details
Flight Controller	Pixhawk 4
Onboard Flight Computer	Raspberry Pi 4
Camera	Raspberry Pi Camera V2 (RaspiCamV2)
GPS	Pixhawk 4 GPS Module
UAV Frame	S500
Motor	Emax XA2212 1400KV 3S Brushless Motor
Battery	4200 mAh
Propeller	10 inches
Flight Altitude	5–15 m
Flight Velocity	Average 2 m/s
Flight Duration	5 min

**Table 4:** RaspiCamV2 characteristics.

Attributes	Details
Size	25 × 24 × 9 mm
Still resolution	8 Megapixels
Video modes	1640 × 1232 and 640 × 480
Sensor	Sony IMX219
Maximum Frame Rate	30 Frame/sec
Pixel size	1.12 µm × 1.12 µm
Focal length	3.04 mm
Horizontal Field of View	62.2 degrees
Vertical Field of View (FoV)	48.8 degrees
Maximum exposure times	11.76
Size	25 × 24 × 9 mm

### 4.2 Critical Safety Protocols for Reliable UAV Data Acquisition

Assuring the integrity of forest fire data collection by UAVs demands rigorous safety protocols to mitigate risks and maintain reliability. This section highlights executing comprehensive efforts during UAV deployment to maintain data integrity and enhance operational efficacy.

#### 4.2.1 Technical Safety Protocols

Proactive protocols were established to mitigate fire hazards associated with UAV batteries. Before deployment, the UAV battery was securely connected to the power distribution board before entering controlled fire zones. Real-time monitoring of the camera's line of presence was facilitated by connecting the Raspberry Pi to a mobile device pre-flight, enabling efficient image assessment without requiring UAV landing. An integrated buzzer system emitted audible warnings to operators upon battery charge insufficiency below a specified threshold, mitigating crash risks.

#### 4.2.2 Operational Safety Protocols

Operational safety benchmarks included comprehensive pre-flight inspections to ensure optimal UAV performance. Inspections covered detailed inspections of battery levels, propeller integrity, and software updates. Flight schedules were carefully planned, considering meteorological conditions to avoid unfavourable weather and ensure UAV safety and data integrity. Adherence to local aviation regulations, including acquiring necessary flight approvals and observation with no-fly zone restrictions, was precisely followed. UAV operators experienced extensive training to ensure proficiency in UAV operation and management, particularly in responding to potential emergencies during flight operations.

### 4.3 Data Preprocessing

#### 4.3.1 Data Augmentation Techniques Applied to Raw Data

Data augmentation, involving transformative operations applied to raw images, enhances dataset diversity and classification accuracy [9,10]. In our study, we employed tkeras.preprocessing.image.ImageDataGenerator from TensorFlow [10,11]. Augmentation parameters were applied, including rotation, width shift, height shift, shear, zoom, horizontal flip, fill mode, and constant value. Rotation adjusts image orientation for real-world scenarios, while width and height shifts vary perspectives and object positions to aid model generalization. Shearing distorts shapes for diverse object angles, and random zooming scales feature robustly. Horizontal flip adds symmetrical representations. Fill mode manages pixel creation during transformations, with 'reflect' Mode used for edges and a constant value of 125 for constant mode. These augmentations enhance performance on unseen data, act as regularizations against overfitting, and improve real-world classification accuracy.

The detail of data augmentation is shown in Table 5. including a 180-degree image rotation, width and height shifts, shearing operations parameterized with a magnitude of 0.2, and a zoom range of 0.30. The batch size is set to 32, with implemented shuffling procedures to mitigate sequence-induced biases. After applying data augmentation techniques, each data class contains 3890 images, resulting in a total augmented dataset of 15,560 instances. This distribution is further detailed in Table 6. After data augmentation we applied the annotation in the augmented images in the Pre-evening fire incident and Evening fire incident classes.

**Table 5:** Details of data augmentation parameters.

Attributes	Parameter
Rotation	180
Width shift	0.2
Hight shift	0.2
Shear	0.2
Zoom	0.3
Horizontal flip	True
Fill mode	Reflect
Size	265×256
Batch size	32

**Table 6:** The number of raw data pre- and post-augmentation.

Classes	Pre-augmentation	Post-Augmentation
Pre-evening Forest condition	222	3890
Evening forest condition	286	3890
Pre-evening fire incident	791	3890
Evening fire incident	354	3890

#### 4.3.2 Challenges and Iterative Refinements in Data Augmentation

Data augmentation is crucial for enriching dataset comprehensiveness and improving classification model performance [9,10]. It involves systematic and iterative refinement amidst complex challenges. A primary difficulty is selecting optimal parameters for each augmentation technique to enhance model robustness without compromising performance integrity [12]. This requires extensive experimentation to effectively balance rotation angles, scaling factors, and flipping probabilities. Additionally, identifying augmentation methods that truly enhance model accuracy remains challenging. Systematically evaluating techniques such as adjusting brightness, enhancing contrast, and adding Gaussian noise are essential for determining transformations that optimally improve model performance during training. This rigorous assessment aims to pinpoint augmentation strategies that deliver the most beneficial outcomes.

### 4.4 Experiment

#### 4.4.1 Multiheaded CNN for forest fire detection (MHCNNFD)

**Fig. 3:**
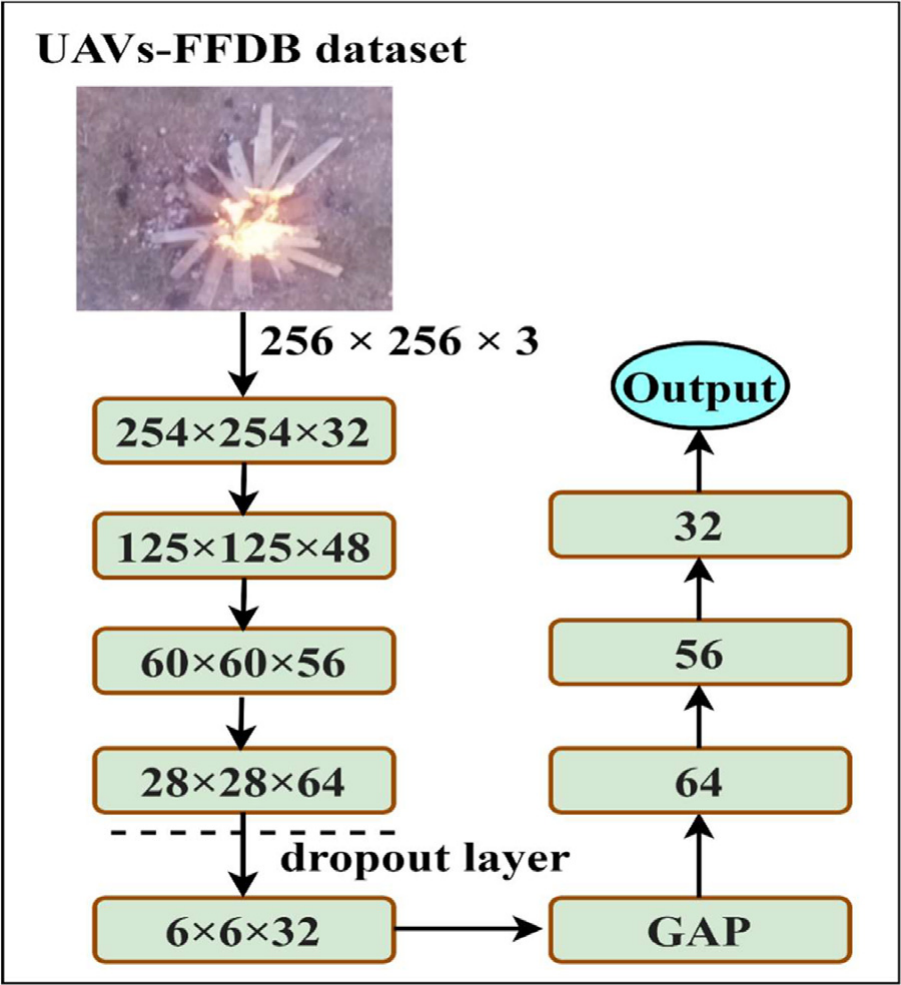
Architecture of MHCNNFD.

This study proposed a lightweight multiheaded convolutional neural network for forest fire detection (MHCNNFD) architecture shown in Fig. 3. The model consists of five convolutional layers for feature extraction and four fully connected layers, each utilizing Karnal size 3×3. The first convolutional layer employs 32 filters, followed by max-pooling with a pool size of 2×2. Subsequently, the second convolutional layer employs 48 filters, followed by max-pooling. This pattern continues with 56 filters in the third convolutional layer, 64 filters, and 32 in the fifth layer. Dropout regularization with a rate of 0.1 is applied after the fourth convolutional layer to mitigate overfitting. Following the convolutional layers, a global average pooling (GAP) layer is utilized to reduce feature map dimensionality. The fully connected layers consist of 64, 56, and 32 neurons, all activated by scaled exponential linear units (SELUs) activation functions. Finally, a SoftMax activation function is employed in the output layer to predict class probabilities. The model is trained employing the Adam optimization algorithm, characterized by a progressive decay in the learning rate (lr) across epochs. Initially, a lr of 1e-3 is applied for the initial 15 epochs, followed by a reduction to 1e-4 from the 16th to the 30th epoch, and further diminished to 1e-5 from the 31th to the 40th epoch. The categorical cross-entropy serves as the loss function. Additionally, a lr scheduler function is incorporated into the training process to dynamically optimize training and adapt the lr throughout the training epochs. The hyperparameter configuration employed within the MHCNNFD architecture is precisely delineated in Table 7.

**Table 7:** Hyperparameter selection of MHCNNFD.

Attribute	Parameter
Input size	256×256
Batch size	32
Activation	SoftMax
Optimizer	Adam
No of epoch	40
Step per epoch	104
Learning rate (lr)	1-14 epoch = 1e-3 15-30 epoch = 1e-4 31-40 epoch = 1e-5
Data separation	80:10:10

The dataset has been stratified by utilizing Sci-kit Learn package [13] into three distinct subsets: a training set, a validation set, and a test set, constituting 80 %, 10 %, and 10 % of the overall dataset, respectively. The training set comprises a total of 12,448 image entries, while the validation and test sets each consist of 1556 images. In this scenario, each class comprises 3112 images allocated for the training, and the distribution for the validation and test set are shown in Table 8.

**Table 8:** Data separation for training, validation and test set for MCNNFD model.

Dataset Classes	Train	Validation	Test
Pre-evening Forest condition	3112	418	360
Evening forest condition	3112	356	422
Pre-evening fire incident	3112	390	388
Evening fire incident	3112	392	386

#### 4.4.2 Experimental Result

The MHCNNFD architecture analyzed a UAV-based forest fire direction dataset, achieving an accuracy of 99.81 % and a loss of 0.0052. It utilized 97,508 parameters and occupied 380.89 KB in size. The training duration was 20 minutes and the prediction time averaged 12 s 8 ms/step for 1,556 images for the testing set. Details, including training, validation, test accuracy, and training and prediction times with no of parameter and size are summarized in Table 9.

**Table 9:** The overall result of the MHCNNFD model.

Training	Validation	Test	Loss	Training time	Prediction time	Parameters	Size
100 %	100 %	99.81 %	0.0052	20 min	12 s 8 ms/step	97508	380.89 KB

**Fig. 4:**
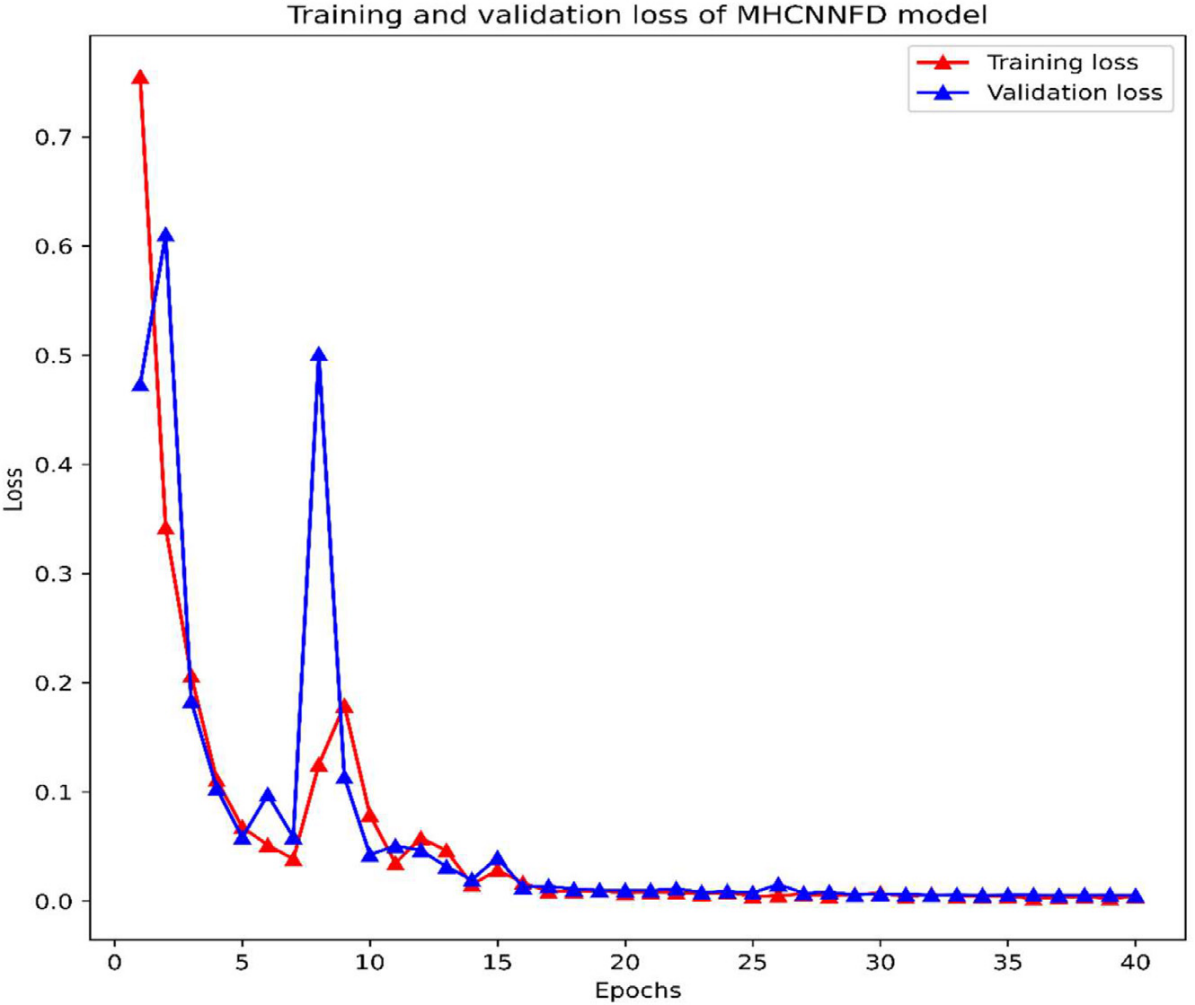
Training and validation loss of the MHCNNFD model.

**Fig. 5:**
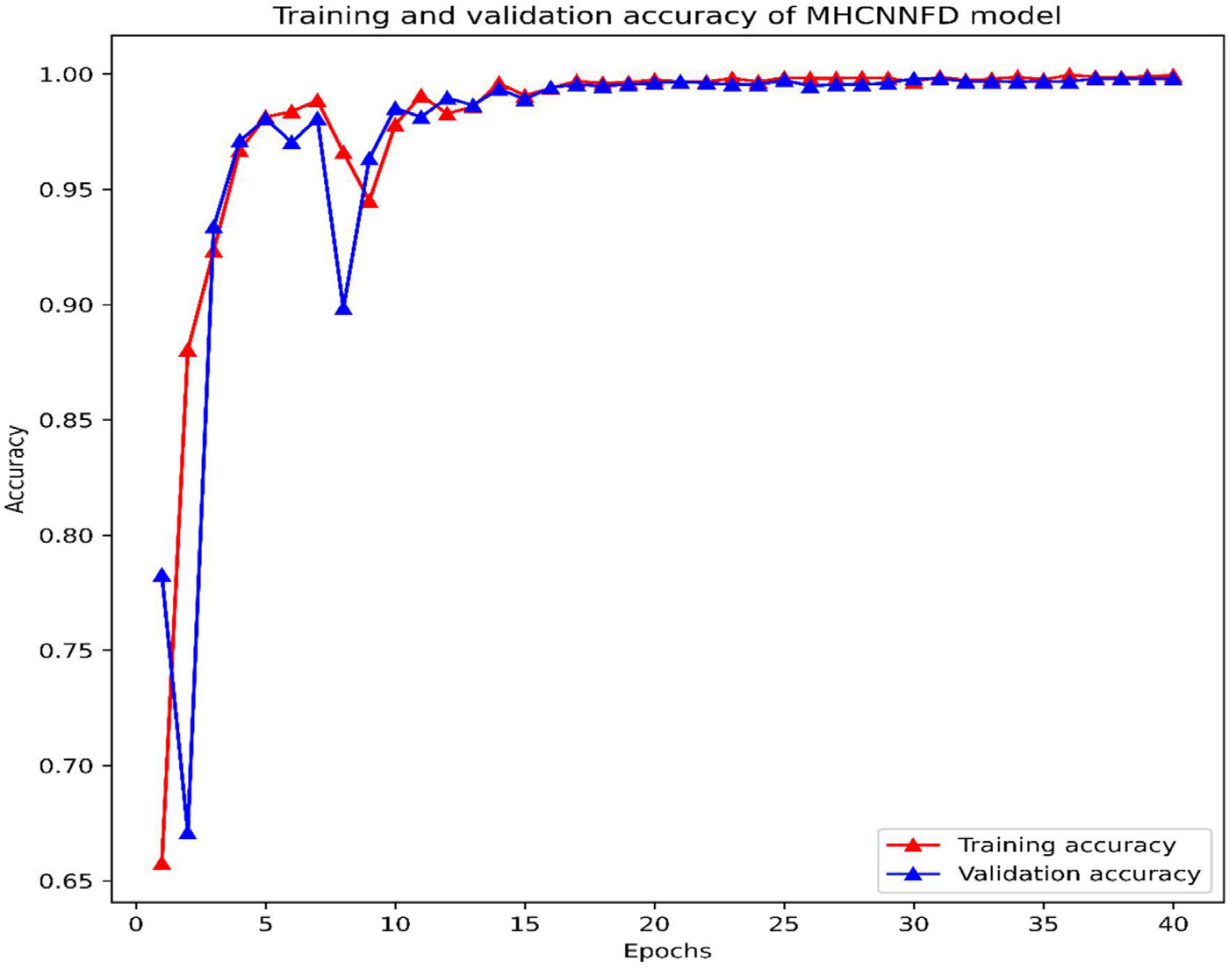
Training and validation accuracy of MHCNNFD model.

The model's performance was assessed using key metrics such as loss and accuracy throughout the training process. The evolution of these metrics throughout training is depicted in Fig. 4, Fig. 5. In the initial epochs, from 1 to 5, the model's loss steadily declined from 0.7551 to 0.0678, accompanied by an increase in accuracy from 65.78 % to 98.14 %. The lr was maintained at 0.0010 during this phase. The validation loss followed a similar downward trend, reaching a minimum of 0.0584 by epoch 5. Following epoch 5, training and validation losses converged, indicating effective generalization without overfitting. This convergence was maintained throughout the subsequent epochs, with losses stabilizing impressively at approximately 0.0067 towards epochs 35 to 40. Simultaneously, the learning rate was reduced to 1e-5. The iterative optimization process facilitated continuous enhancement of the model's performance and generalization capabilities. By the end of the training, the model achieved a remarkable accuracy level of approximately 99.81 %.

#### 4.4.3 Evolution matrix

Our analysis focused on four fundamental metrics to construct the predictive model: accuracy, precision, recall, and F1-score. The term true positive denotes the fraction of correctly identified positive outcomes (TP). Conversely, false negatives represent instances where predictions fail to identify positive outcomes (FN). False positives occur when the model incorrectly identifies an outcome as positive (FP). Lastly, true negatives refer to accurately predicted negative outcomes (TN). These metrics collectively provide a comprehensive evaluation of the model's predictive performance, enabling a nuanced understanding of its strengths and limitations.

(1) $$\text{Accuracy}=\;\frac{TP+TN}{TP+FP+TN+FN}$$

(2) $$\text{Precision}=\;\frac{TP}{TP+FP}$$

(3) $$\text{Recall}=\;\frac{TP}{TP+TN}$$

(4) $$F1\;\text{score}=\;\frac{2\times Precision\;\times Recall}{Precision+Recall}$$

The classification report, delineating performance metrics for each class within the dataset illustrated in Table 10, is presented herein. For the Pre-evening Forest condition class, the model attained a precision and F1-score of 100 %, alongside a recall rate of 99 %. Similarly, in the Evening Forest condition class, all metrics achieved perfection at 100 %, denoting precise identification of instances pertaining to both pre-evening and evening forest conditions. Within the Pre-evening fire incident class, the model showcased a precision of 99 % and achieved a recall rate and F1-score of 100 %. Similarly, the Evening Fire Incident class achieved 100 % precision, recall, and F1-score. The overall accuracy, serving as a metric for the proportion of correctly classified instances, was documented at 99.81 %. Notably, each class was supported by varying numbers of images, specifically 388, 386, 360, and 422 images for the Pre-evening Forest condition, Evening Forest condition, Pre-evening Fire Incident, and Evening Fire Incident classes, respectively.

**Table 10:** Classification report of the MHCNNFD architecture.

Classes	Precision	Recall	F1 score	Support
Pre-evening Forest condition	100 %	99 %	100 %	778
Evening forest condition	100 %	100 %	100 %	778
Pre-evening fire incident	99 %	100 %	100 %	778
Evening fire incident	100 %	100 %	100 %	778
Accuracy			99.81 %	

**Fig. 6:**
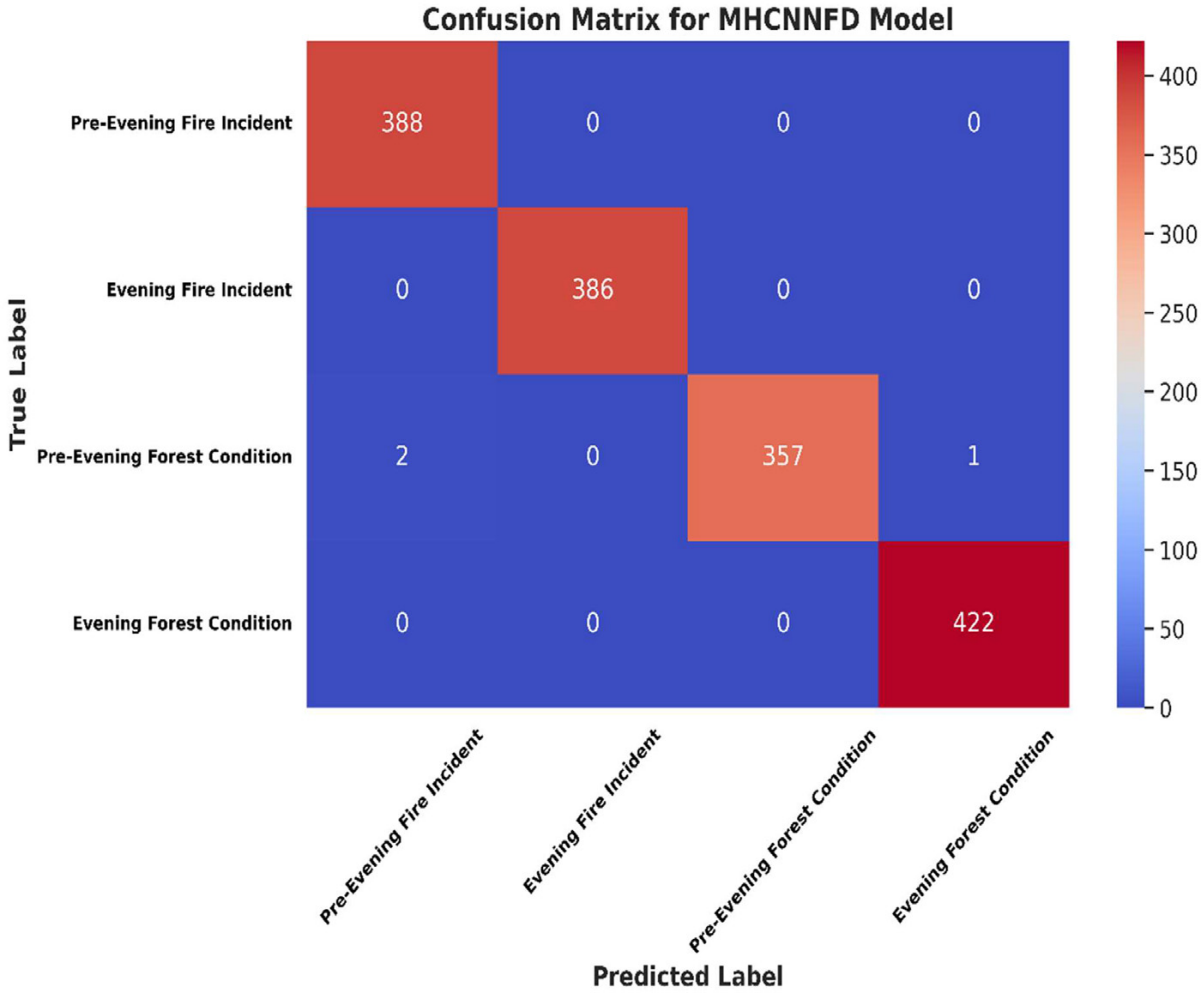
Confusion matrix for MHCNNFD architecture.

Fig. 6 presents the confusion matrix generated by the applied model. The confusion matrix reveals excellent predictive accuracy for the classes Pre-Evening Fire Incident and Evening Fire Incident, with 388 and 386 instances correctly classified, respectively. Similarly, the model demonstrates high accuracy for the classes Pre-Evening Forest Condition and Evening Forest Condition, with 357 and 422 instances correctly classified, respectively. Despite overall high accuracy, there are instances of misclassification between closely related classes. Specifically, there are two instances where Pre-Evening Fire Incidents are misclassified as Pre-Evening Forest Conditions, and one instance where a Pre-Evening Forest Condition is misclassified as Evening Forest Condition. No misclassifications were observed between instances of Evening Fire Incident and other classes.

Several strategies can be utilized to mitigate these misclassifications and enhance model accuracy. Fine-tuning the feature selection and integrating techniques such as the attention mechanism and updating the layers with filter size and hyperparameter can help determine more effectively between identical classes by emphasizing subtle differences in the data. Besides, executing state-of-the-art ensemble approaches, such as stacking or boosting, could enhance classification performance by combining the strengths of multiple models. Additionally, increasing the size and diversity of the training dataset may provide the model with a more overall range of examples, thereby enhancing its ability to distinguish between closely related classes. Eventually, integrating domain-specific knowledge by incorporating expert features or rules can extend the model's discrimination capability. These techniques aim to facilitate representatives of misclassification and ensure more reliable predictions in forest fire detection using UAVs.

**Fig. 7:**
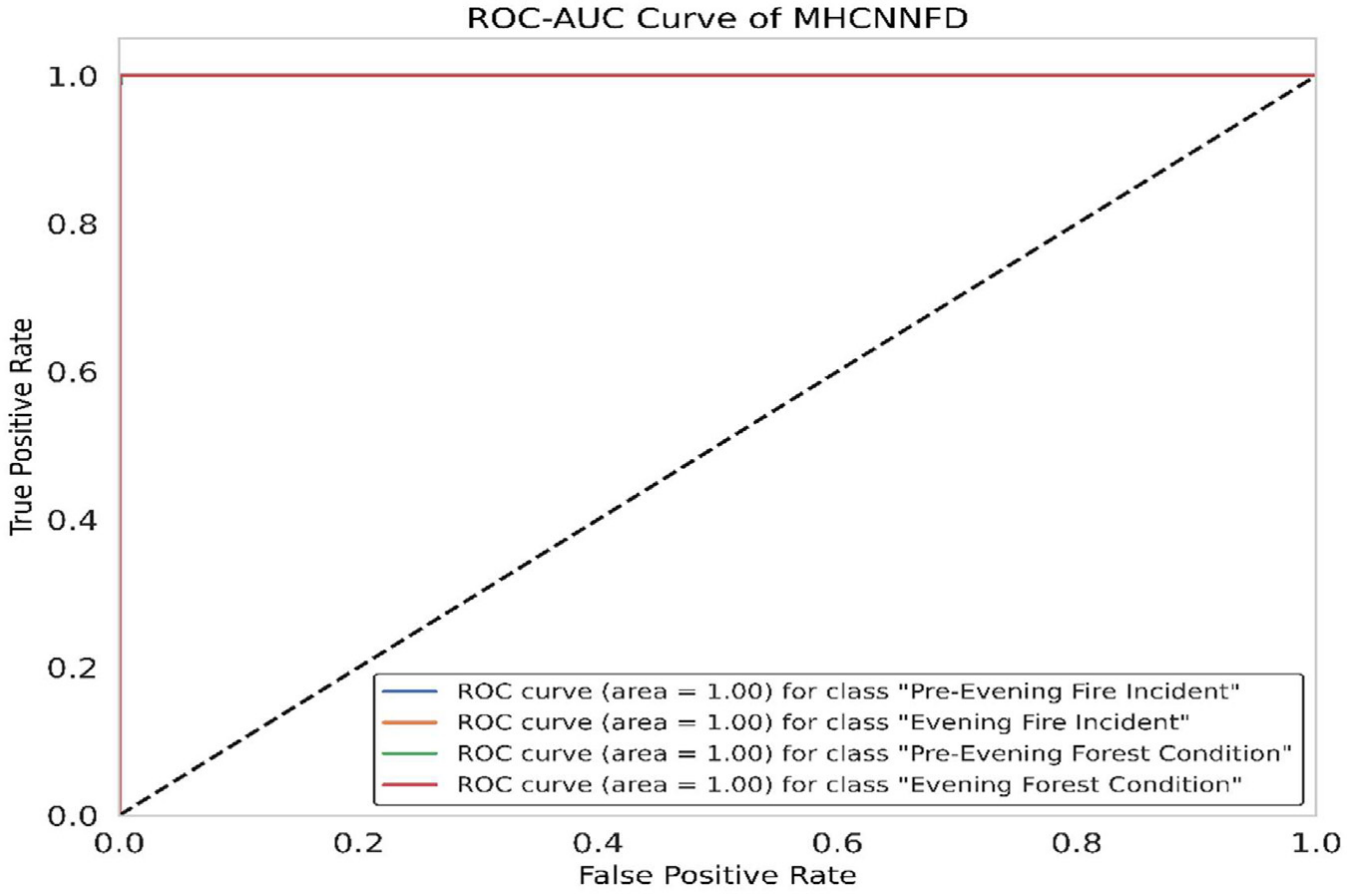
ROC-AUC for MHCNNFD model.

The ROC-AUC for the MHCNNFD model is demonstrated in Fig. 7. A score of 1 for each class indicates perfect discrimination ability, demonstrating optimal performance in determining between Pre-Evening Fire Incident, Evening Fire Incident, Pre-Evening Forest Condition, and Evening Forest Condition. This suggests the effective utilization of features for accurate classification across all classes, emphasizing its potential for robust application in fire incident prediction and forest condition assessment.

#### 4.4.4 Comparison with Existing Research

Recent advances in forest fire detection have resulted in several significant studies, predominantly utilizing datasets sourced from the internet or open-access repositories, complemented by limited real-world imagery obtained via UAVs. The research discusses based on recent data collection of forest fire by UAVs, detailed in Table 11. It is noteworthy that the FLAME [13] and Fire datasets [14], collected in 2021 and 2017 respectively, were specifically obtained via UAVs in forest environments. In contrast, the remaining datasets utilized in this study were sourced from open-access repositories available on the internet, i.e., the Fire_Seg dataset [15] and the Yar dataset [16].

**Table 11:** Comparison of existing and proposed datasets using advanced methodologies.

References	Dataset	Source	Classes	DL Model	Accuracy
[11]	FLAME [13]	O/A	Fire; No-Fire	InceptionV3, DenseNet121, ResNet50V2, NASNetMobile, VGG-19, SVM, RF, Bi-LSTM, GRU	97.95 %
[6]	FLAME[13]; Fire_Seg [15]	O/A	Fire; No-Fire	ADE-Net	80.25 %; 83.80 %
[17]	FLAME [13]	O/A	Fire; No-Fire	Attention-EfficientNetB0	92.02 %
[4]	Fire dataset [14]	O/A	Fire; No-Fire	FedSGD	99.27 %
[16]	Foggia's; FD; Yar [16], complex fire dataset	O/A, Self	Fire; No-Fire	ViT	81.61 %
This study	UAVs-FFDB	Self	Pre-evening forest; evening forest; pre-evening fire; evening fire	MHCNNFD	99.81 %

Reis et al. proposed multiple deep learning models for forest fire detection using the FLAME dataset, encompassing thermal (fusion, white-hot, green-hot) and normal spectrum images. This dataset features videos at 29/30 FPS and 3840×512 resolution (mp4, mov), captured using specialized Thermal, Full HD, and 4K cameras mounted on UAVs, partitioned into training (14,357 no-fire and 25,018 fire instances) and test sets (3,480 no-fire and 5,137 fire instances). Various DL algorithms, including InceptionV3, DenseNet121, ResNet50V2, NASNetMobile, and VGG-19, were employed alongside transfer learning and hybrid methodologies integrating Support Vector Machine (SVM), Random Forest (RF), Bidirectional Long Short-Term Memory (Bi-LSTM), and Gated Recurrent Unit (GRU) models. DenseNet121 achieved 97.95 % accuracy with random weights and 99.32 % accuracy with ImageNet weights during transfer learning [11]. Similarly, Kong et al. introduced an innovative pixel-wise fire flame area extraction network (ADE-Net) applied to FLAME and Fire_Seg datasets, the latter comprising images extracted from 20 YouTube videos depicting outdoor fire scenes under diverse lighting conditions. ADE-Net achieved a Dice coefficient of 80.25 % and a mean Intersection over Union (mIOU) of 83.80 % [6]. Furthermore, Aral et al. presented models incorporating transfer learning, deep CNNs, and lightweight CNNs applied to the FLAME dataset, with the attention-based EfficientNetB0 model emerging as the most successful, achieving a test accuracy of 92.02 %, thus affirming its effectiveness in wildfire recognition [17].

Siddique et al. introduced a novel framework utilizing Federated Stochastic Gradient Descent (FedSGD) and Internet of Things (IoT) technology to improve fire detection. They applied this method to a Kaggle fire dataset distinguishing Fire and No-Fire classes, achieving 99.27 % accuracy [4]. Concurrently, Yar et al. utilized vision transformers across four datasets: Foggia's (31 videos, 14 with fire scenes), FD (benchmarking Foggia's and BoWFire datasets with fire and normal categories), Yar (addressing fire-like colors with 1000 forest-fire and 1000 non-fire images), and a complex fire dataset (7642 images capturing various fire scenarios, including 4036 fire incidents). The models achieved an average accuracy of 81.61 % across these datasets, demonstrating their efficacy in fire detection tasks [16].

This study extends existing research by developing the UAVs-FFDB dataset, which consists of real images captured by UAVs in forest environments. The previous datasets especially included binary classes (fire and no-fire) and depended on open-access internet sources, which have been discussed in above. The UAVs-FFDB dataset comprises four classes, accommodating variations in image and lighting conditions (including day and night scenarios). This comprehensive dataset addresses prior limitations by providing a more diverse and realistic set of images for training and evaluation.

In addition, the proposed MHCNNFD model demonstrates superior performance on this dataset and achieved 99.81 % accuracy. Evaluation of this model indicates that the UAVs-FFDB dataset-driven approach achieves outstanding accuracy compared to conventional and existing methods applied to the FLAME, Fire_Seg, Foggia, and Yar datasets as shown in Table 11. Specifically, the MHCNNFD architecture achieved higher accuracy rates, confirming the efficacy of our dataset and methodology. Furthermore, the findings underscore the importance of utilizing real-world, diverse datasets like UAVs-FFDB for enhancing the accuracy and reliability of forest fire detection systems. The MHCNNFD model, driven by this dataset, significantly improves existing methods, providing a robust tool for early fire detection and management.

## 5 Challenges and Dataset Limitations

Forest fire detection dataset utilizing UAVs presents considerable technological, functional, and environmental challenges. Unfavorable weather conditions, including extreme winds, rainfall, and haze, restrain field operations and complicate the preference for controlled burn surroundings. In addition, it affects UAV stability, sensor functionality, and data accuracy. Technological challenges in dataset enlargement warrant insistent advancements, while safety protocols limiting fire ignition in dense forest areas further hinder data collection. Limited battery life restricts UAV flight durations, requiring multiple charges for adequate data acquisition. The complexity of forest landscapes and dense canopies obstructs UAV navigation, complicating fire detection efforts. UAVs face sensor resolution and range conditions, which affect detailed data collection and real-time transmission, particularly in regions with poor remote connectivity. Besides that, the operational challenges possess collisions with barriers and potential damage near active fires due to high temperatures and turbulent air currents. Data processing requires significant storage and computational resources for accuracy through training and validation against base data. Given the rapid spread of fires, personnel with fire suppression tools must accompany UAV operations. Additionally, the substantial proceeding costs associated with UAVs and various sensor and hardware systems pose significant financial challenges in establishing and maintaining these datasets for scientific research.

## 6 Technical Discussion and Future Works

This section examines the experimental implications of our UAV-acquired high-resolution forest fire detection dataset. Integrating this dataset with UAV systems advances forest fire management, enhancing proactive prevention and response strategies.

• **Integration with UAV Systems**

The high-resolution forest fire detection dataset, designed for integration with UAVs, marks a significant advancement in forest fire management [11,13]. It enhances aerial surveillance with detailed spatial and temporal data on fire areas, optimizing UAV flight paths and data collection strategies for improved fire prevention and response [13]. Real-time data transmission capabilities facilitate rapid, timely interventions.

• **Data Processing Technique**

The methodology uses data processing algorithms to examine UAV-acquired imagery. These algorithms utilize data augmentation techniques, including rotation, zooming, scaling, flipping, and controlled value adjustments to provide early fire detection, fire propagation analysis, and assessment of fire-conducive environmental conditions.

• **Enhanced Detection Accuracy**

Empirical evaluations show that our dataset-driven approach offers superior accuracy compared to traditional methods. UAVs equipped with this dataset swiftly identify and localize potential fire incidents by capturing details such as smoke plumes, thermal signatures, and vegetative health indicators. This reduces response times, mitigating wildfire escalation and associated risks.

• **Scalability and Practical Implementation**

The applied model and its evaluation process indicate that the UAVs-FFDB dataset-driven approach presents outstanding accuracy compared to conventional methods applied to the FLAME, Fire_Seg, Foggia, and complete datasets. UAVs with this dataset recognize and localize potential fire incidents by capturing details such as smoke plumes, thermal signatures, and vegetative health indicators. This reduces response times, mitigating wildfire expansion and associated risks.

• **Application and Use Cases**

The UAVs-FFDB dataset, while primarily designed for forest fire detection, offers substantial potential for a diverse range of applications. Its high-resolution imagery and comprehensive annotations make it suitable for general object detection tasks, leveraging the detailed and varied data provided. Beyond object detection, the dataset is invaluable for considerable environmental monitoring activities. For instance, it facilitates wildlife monitoring by enabling the detection and tracking of animal movements, thereby supporting conservation efforts [2]. The UAVs-FFDB dataset also enhances land cover classification, aiding in the mapping and managing of natural resources [1]. Furthermore, it holds significant promise for agricultural management, enabling precise crop health monitoring and yield prediction [18,19]. By encompassing these diverse use cases, the UAVs-FFDB dataset emerges as a universal resource, advancing research and practical applications in environmental science and beyond.

• **Challenges and Future Directions**

Despite UAVs-based forest fire detection effectiveness under controlled conditions, ongoing research is essential to address challenges such as cloud cover variability, landscape complexity, and sensor calibration distinctions. Future research aims to expand dataset coverage, integrate multi-sensor data fusion methodologies [18], and improve predictive architecture and decision support systems. Innovations in data compression techniques and optimized UAV deployment protocols [19,20] are required to overcome bandwidth limitations of wireless network protocols [20] and improve operational persistence, maximizing the potential of real-time monitoring.

## Ethics Statement

Our study does not involve studies with animals or humans. Therefore, we confirm that our research strictly adheres to the guidelines for authors provided by Data in terms of ethical considerations.

## CRediT authorship contribution statement

**Md. Najmul Mowla:** Conceptualization, Data curation, Formal analysis, Investigation, Methodology, Software, Validation, Visualization, Writing – original draft, Writing – review & editing. **Davood Asadi:** Data curation, Funding acquisition, Project administration, Supervision, Writing – review & editing. **Kadriye Nur Tekeoglu:** Data curation. **Shamsul Masum:** Supervision, Writing – review & editing. **Khaled Rabie:** Writing – review & editing, Funding acquisition.

## Data Availability

UAVS-FDDB: UAVs-based Forest Fire Detection Database (Original data) (Mendeley Data).
